# Supplementary material for: Correction: Honey Bee Hemocyte Profiling by Flow Cytometry
Source: PLoS One. 2014 Dec 16;9(12):e116148. doi: 10.1371/journal.pone.0116148 (PMC4267832; doi:10.1371/journal.pone.0116148)
Supplement: File S1 — Originally published, uncorrected article. (PDF) [file pone.0116148.s001.pdf]

OPEN ACCESS Freely available online

PLOS ONE

Honey Bee Hemocyte Profiling by Flow Cytometry

William J. Marringa, Michael J. Krueger, Nancy L. Burritt, James B. Burritt*

Department of Biology, University of Wisconsin-Stout, Menomonie, Wisconsin, United States of America

Abstract

Multiple stress factors in honey bees are causing loss of bee colonies worldwide. Several infectious agents of bees are believed to contribute to this problem. The mechanisms of honey bee immunity are not completely understood, in part due to limited information about the types and abundances of hemocytes that help bees resist disease. Our study utilized flow cytometry and microscopy to examine populations of hemolymph particulates in honey bees. We found bee hemolymph includes permeabilized cells, plasmatocytes, and acellular objects that resemble microparticles, listed in order of increasing abundance. The permeabilized cells and plasmatocytes showed unexpected differences with respect to properties of the plasma membrane and labeling with annexin V. Both permeabilized cells and plasmatocytes failed to show measurable mitochondrial membrane potential by flow cytometry using the JC-1 probe. Our results suggest hemolymph particulate populations are dynamic, revealing significant differences when comparing individual hive members, and when comparing colonies exposed to diverse conditions. Shifts in hemocyte populations in bees likely represent changing conditions or metabolic differences of colony members. A better understanding of hemocyte profiles may provide insight into physiological responses of honey bees to stress factors, some of which may be related to colony failure.

Citation: Marringa WJ, Krueger MJ, Burritt NL, Burritt JB (2014) Honey Bee Hemocyte Profiling by Flow Cytometry. PLoS ONE 9(10): e108486. doi:10.1371/journal.pone.0108486

Editor: Gro V. Amdam, Arizona State University, United States of America

Received February 14, 2014; **Accepted** August 29, 2014; **Published** October 6, 2014

Copyright: © 2014 Marringa et al. This is an open-access article distributed under the terms of the Creative Commons Attribution License, which permits unrestricted use, distribution, and reproduction in any medium, provided the original author and source are credited.

Funding: The authors wish to acknowledge funding from the Honey Bee Health Task Force of the North American Pollinator Protection Campaign (NAPPC). The authors also acknowledge an internal Student Research Grant from the University of Wisconsin-Stout Access to Learning Program and a grant from the Stout University Foundation. The opinions expressed are those of the authors and do not necessarily reflect the views of the funders. The funders had no role in study design, data collection and analysis, decision to publish, or preparation of the manuscript.

Competing Interests: The authors have declared that no competing interests exist.

* Email: burrittj@uwstout.edu

Introduction

Overview

The western honey bee (*Apis mellifera*) contributes to about one third of the food supply for humans [1]. Commercial-scale production of almonds, certain fruits (apple, apricot, peach, and cherry) and some vegetables (cucumbers and melons), would not be possible without their role in pollination. In recent decades, honey bee colonies have declined in most agricultural areas worldwide. During the 2012–2013 season, colony loss for the average US beekeeper was 44.8% [2], with increasing concern regarding bee health also in Europe and other locations [3,4]. This situation threatens the global food supply for an expanding human population. The cause for this loss appears to be multifactorial [5] and has defied clear definition.

Considerable effort is being devoted to understanding threats that impact normal function of honey bee colonies. Pathogens such as *Varroa destructor* mites [6], tracheal mites [7], two species of *Nosema* intestinal parasites [8,9], bacteria [10], fungi [11], and viruses [12–14] are now recognized to infect honey bees and threaten their survival [15–18]. Application of xenobiotics (pesticides, herbicides, and fungicides) has also been implicated in the decline of honey bee colonies [19–21]. In addition, climate change, variability in nutritional sources for bees [11,22], and trends toward migratory beekeeping create additional stress on managed hives [4,23]. Our ability to mitigate stress factors of honey bees will require a better understanding of their defense and response mechanisms. At this time, however, few metrics are available upon which changes in honey bee metabolism can be evaluated and understood.

Honey bee immunity

The immune systems of insects have some similarities with innate defense strategies in mammals [24], which can be broadly separated into cellular and humoral (soluble) components. When compared with other insects such as *Drosophila* and *Anopheles*, honey bees have only about one third the number of genes devoted to immunity, suggesting either their immunologic efficiency, or vulnerability to infection [25]. Insect hemocytes are a central component of their cellular host defense, wherein mechanisms of phagocytosis, nodulation, encapsulation, and melanization have been described [26,27]. Despite the importance of honey bee hemocytes in resisting disease and several fruitful studies involving this topic [28–34], a number of details about cell types, numbers, and response to challenge are lacking. Therefore, one goal of our study was to extend the work of others who have shown differences between hemocyte types in honey bees.

Honey bees require complex immune defense mechanisms. When compared with solitary insects, they may utilize additional strategies that limit spread of infection through close contact in society members [35–38]. Also, considerable interest has focused on the possibility that honey bees sacrifice or suppress some aspects of immune defense in their later adult life, in exchange for other capabilities [10,29–31,39–41]. Even as many of these details continue to emerge, it is now evident that bee colonies that succumb to infectious agents herald mechanisms of disease that breach natural immune surveillance and control.

Hemocyte subsets

Examination of insect hemocyte subsets has been carried out in several species, including the fruit fly (*Drosophila melanogaster* [42]), mosquito (*Aedes aegypti* [43]), and silkworm, (*Bombyx mori* [44–46]); as well as in a variety of other invertebrates, including shellfish [47], arachnids [48], crustaceans [49], and ascidian tunicates [50]. Fundamental differences in hemocyte types, combined with a paucity of probes for specific cell types, has hindered development of a classification scheme that can be broadly applied across insect orders [26,51]. We hypothesize that in honey bees, stimuli from infectious agents, xenobiotics, nutritional changes, migratory beekeeping practices, seasonal variation, age, and social caste may be reflected in the abundance and types of their hemocytes. A refined understanding of cellular immunity in honey bees could provide new views on their metabolic responses to a spectrum of challenges; some of which threaten their survival.

Our claim

Our study was carried out to contribute to a better understanding of the circulating immune cell types in honey bees. We utilized a combination of flow cytometry and microscopy to describe the subsets of particles in bee hemolymph. Based on comparison to the Diptera, Lepidoptera, and other members of the Hymenoptera insect orders, we observed three primary types of hemocytes in honey bees: 1) permeabilized cells (of uncertain origin) displaying plasma membranes permeable to propidium iodide (PI) and bound by wheat germ agglutinin-FITC (WGA) and annexin V-FITC (annexin V), 2) a similar subset of permeabilized cells that differed in the fact that they were not bound by WGA and had contrasting light scatter properties, and 3) plasmatocytes containing protracted nuclei not labeled by PI, and intact plasma membranes not bound by annexin V, but variably bound by WGA. Both sets of permeabilized cells and the plasmatocytes failed to reveal mitochondrial membrane potential when probed by the JC-1 fluorochrome, which has previously been shown to label active mitochondria in permeabilized cells from larvae of the lepidopteran insect, *Spodoptera frugiperda* [52]. The most numerous hemolymph objects were under 3 μm in diameter, did not contain nuclei, and resembled microparticles or membrane blebs, which have not previously been described in honey bees.

We found hemolymph particulates could be efficiently differentiated based on labeling by PI and WGA. Using flow cytometry, fluorescent particle information could be generated for individual bees, and averaged for a select group. When analysis was applied to bees from a single colony, significant differences between individuals were noted. Therefore, this approach may be used to learn more about polyethism, polyandry, or physiology of individual bees, and potentially their responses to external stimuli.

To demonstrate how hemocyte analysis could be utilized to study the impact of a stress factor on honey bee populations, we applied this approach to colonies that differed in the numbers of associated *Varroa* mites. Distinctions in both total numbers and fluorescent WGA labeling intensity of plasmatocytes were noted among bees from these colonies. Our results suggested hemolymph indices are reactive to certain stimuli that impact colony health, and may provide a new means of evaluating select physiological parameters in hive members.

Materials and Methods

Ethics Statement

Studies did not involve endangered or protected species. Following obtaining permission from the owners of the private apiaries, approximately 120 honey total honey bees were collected from a private apiary in River Falls Township in Pierce County, Wisconsin and approximately 1500 total were collected from a private apiary in Tainter Township in Dunn County Wisconsin.

Reagents

Unless indicated otherwise, all reagents and chemicals were obtained from either Fisher Scientific (Waltham, MA, USA) or Sigma Aldrich (St. Louis, MO, USA). *Honey bees:* Colonies of honey bees were housed in standard 10-frame, deep-body Langstroth hives. Bees were collected from the top edge of honey combs, above and away from the brood-rearing area. This location was chosen for consistency, and to reduce the chance of collecting hatchlings and nurse bees which may represent different phenotypes of hemocyte subsets. We consulted a reference for standard methods in honey bee research [53] for guidance on hive and bee sampling and for anesthesia of bees by chilling. Our studies included flow cytometry and Wright-stained slides for microscopy involving 25 Carnolian bees from one hive, 25 Russian bees from another hive, 416 Buckfast-Italian cross bees from three separate hives, and 168 Italian bees from two additional hives, providing a total of 634 individual bees. Our analyses included different bee strains to represent some of the more common managed genetic lines. Data produced by these experiments did not identify strain-specific differences in hemocytes. A greater number of hives might be needed to investigate that possibility in detail. Examination of hemocyte subsets in bees with respect to numbers of *Varroa* mites was restricted to three colonies of Buckfast-Italian bees housed in modern equipment purchased from Mann Lake Bee Company in Hackensack, MN.

For all analyses, bees were immobilized in plastic 500 ml bottles by cooling on wet ice for at least 30 minutes [53]. A 26-guage hypodermic needle was then used to puncture the dorsal surface of the abdominal cuticle between the second and third tergite, and 2-3 μl of hemolymph was extracted using a micropipette.

Smears of all specimens were prepared and examined to obtain microscopic information of hemocytes and to screen samples for contamination by gut contents. Glass slides were first coated with 0.1% gelatin (EmbryoMax # ES-006-B, EMD Millipore Corporation, Billerica, MA, USA), and after 10 minutes excess gelatin solution was aspirated and slides were air dried. About 0.5 μl of raw hemolymph was then spread on a small area of the coated slide and air dried prior to Wright staining (Diff-Quik; Dade Bering, Newark, DE, USA) according to the direction of the manufacturer. Samples that suggested contamination with gut contents (due to honey that did not dry prior to staining, or pollen grains that were evident by microscopy) were excluded from further analysis. Two additional microliters of hemolymph were diluted 100-fold in Hanks Balanced Salt Solution (HBSS #10-547F Cambrex Bio Science, Walkersville, MD, USA) containing 1.5 μM PI (ICN19545825, MP Biochemicals, Solon, OH, USA) and 100 ng/ml (2.6 nM protein) WGA-FITC (Sigma, L4895). The cells were then incubated for 15 minutes at 37°C, mixed, and examined immediately (without centrifugation) by flow cytometry.

Flow Cytometry

A Millipore EasyCyte 5 flow cytometer (using a laser excitation wavelength of 488 nm and Millipore GuavaSoft InCyte 2.6 software) was used to examine samples. A bead set standard provided by Millipore was monitored periodically during the study to ensure reproducibility of scatter and fluorescence measurements, and found to be within all tolerances set by the manufacturer. Red and green fluorescence values were used to evaluate particulate binding by PI and WGA (or annexin V),

respectively, and to determine fluorescence following JC-1 staining. A total of 5000 events were collected from each bee or sample, and a threshold value of 25 in forward scatter (FSC) was typically applied to prevent data collection for smaller particles. Both total cell counts and cell percentages were obtained by flow cytometry software. In some cases, flow cytometric analysis was applied to a specific cell type by first gating on the cell population in FSC vs side scatter (SSC) plots, and then determining the mean fluorescence intensity values for the selected population. In this way, fluorescence measurements and concentrations for a given cell type could be determined. For certain analyses, hemolymph probed with PI and WGA as described above was treated with formalin by adding 0.3 volumes of 10% phosphate buffered formalin, (Fisher Scientific, #SF100-4) at least 15 minutes prior to analysis by flow cytometry.

Apoptosis analysis by annexin V and JC-1

Hemocytes were collected from bees, then probed for molecular evidence of apoptosis. Samples were first exposed to PI and annexin V to evaluate plasma membrane permeability and phosphatidylserine accessibility in the presence of calcium (using the #APOAF annexin V apoptosis detection kit from Sigma). Next, hemocytes were tested for mitochondrial membrane potential using JC-1 (Mitoprobe kit #M34152, Molecular Probes of Life Technologies, Carlsbad, CA), in both the presence and absence of the mitochondrial membrane potential disruptor, carbonyl cyanide 3-chlorophenylhydrazone (CCCP) prior to analysis by flow cytometry. Permeabilized cells and plasmatocytes were examined separately for evaluation of labeling by PI and annexin V or JC-1 by selecting specific cell populations from FSC vs SSC plots. As a positive control for the JC-1 analysis, log-phase murine SP2/0 cells were examined in parallel with hemocytes. At least seven different samples containing 5 bees each were prepared and examined for the annexin V and JC-1 analyses. For both annexin V and JC-1 staining, samples were washed in buffer immediately prior to flow cytometry analysis.

Hemocyte classification

Honey bee hemocyte subsets were examined and compared to results from several different insect species. These reports included honey bees [28], certain members of the Diptera and Lepidoptera insect orders (as described in reviews by Lavine and Strand [26], as well as by Ribeiro and Brehélin [51]), and fluorescence-labeling analysis of hemocytes from *Bombyx mori* [45,46]. Additional information on hemocyte subset identification was derived from the Hymenoptera stingless bee, *Melipona scutellaris* [54].

Estimation of *Varroa destructor* mite numbers in hives

Adhesive papers were placed under screened bottom boards of hives to quantify falling *Varroa* mites. The mites were then counted approximately every 2 weeks in three Buckfast-Italian hives over a 16-week period (beginning August 1, 2013, day = 0). Mite counts were then averaged for the number of elapsed days for each collection, and reported as the number of mites collected per day.

Microscopy and Statistical Analysis

All microscopic examinations were performed on a Zeiss Axioscope 2-Plus microscope and imaging system using Zeiss Axiovision software. All photomicrographs were obtained using a Zeiss 100× planapochromat oil immersion objective (numerical aperture 1.4), creating a total magnification of 1000×. Levene's test for homogeneity of variance and a Shapiro-Wilk test for normal distribution were applied to data sets before examination by ANOVA. If the overall P value was below 0.05, pairwise comparisons were made in a Tukey analysis. Mean values are given as ± standard error of the mean. In all analyses, P values of <0.05 were considered significant. Linear regression analysis was performed to determine the significance of correlation between the FSC (size) and green fluorescence (WGA-FITC fluorescence) of plasmatocytes, and again to test for correlation between the number of *Varroa* mites collected per day from three hives to the number of plasmatocytes in bees from those hives. Statistical analyses were performed using R software (version 3.0.2) or GraphPad Prism (version 3.03).

Results

Flow cytometric analysis of honey bee hemolymph

Flow cytometry analysis of hemolymph from a single representative bee following exposure to PI and WGA revealed four primary particle subsets (Figure 1A). Using unlabeled samples, we ruled out unexpectedly high natural fluorescence in honey bee hemocytes that could be interpreted as specific labeling (data not shown). In one particular bee, quadrants 1-4 (Q1-Q4) were populated by events with percentages of 11/19/27/43, respectively, as shown in Figure 1A. This numbering system that provides relative or quantitative data for particles in the four fluorescence quadrants will hereafter be referred to as the hemocyte profile in our study. Differential coloring of the fluorescent quadrant events shown in Figure 1A was used to identify the corresponding particles from the same sample in the FSC vs SSC plot (representing particle size and granularity, respectively) shown in Figure 1B. The average total hemolymph particle concentration for members of the colony from which the bee represented in Figure 1A and B originated, was determined by flow cytometry to be 4.12×10^4 particles/µl ($\pm8.0\times10^3$ particles/µl, n = 16).

Relationships between fluorescence and light scatter properties for hemolymph particles are illustrated in Figures 1A and 1B. Rationale for identification of particles is explained below in "Hemolymph particle identification". Hemocytes that appeared in the upper left quadrant (Q1, yellow dots) of Figure 1A were identified as the first of two subsets of permeabilized cells. These Q1 cells showed obvious staining by PI but not WGA. Light scatter values (FSC vs SSC) suggested the Q1 permeabilized cells were intermediate in both size and optical complexity relative to other hemolymph particles. The majority of events from the upper right quadrant (Q2, red dots) were determined to be the second subset of permeabilized cells. We observed that about half of the particles in the Q2 quadrant could be shifted to the Q1 quadrant by treatment in buffered formalin for 15 minutes as described in the Methods (data not shown). Cells appearing in Q2 were stained not only by PI, but also by the WGA plasma membrane probe. They also demonstrated higher FSC and SSC values when compared with permeabilized cells in Q1, indicating they have greater size and granularity. A minor set of particles in Q2 showed particularly strong signal in both the fluorescence and light scatter channels, which could represent either a rare cell type or particle aggregate. Microparticles not bound by either fluorescent probe appeared in the lower left quadrant (Q3, blue dots) of Figure 1A, and typically mapped low for FSC and SSC, which suggested their small size and low granularity as shown in Figure 1B. We identified plasmatocytes in the lower right quadrant (Q4, green dots in Figure 1A), which were bound by WGA but not PI. These Q4 cells formed a clear group in the FSC vs SSC plot (Figure 1B) where they were found to be the largest (in dimension) of the

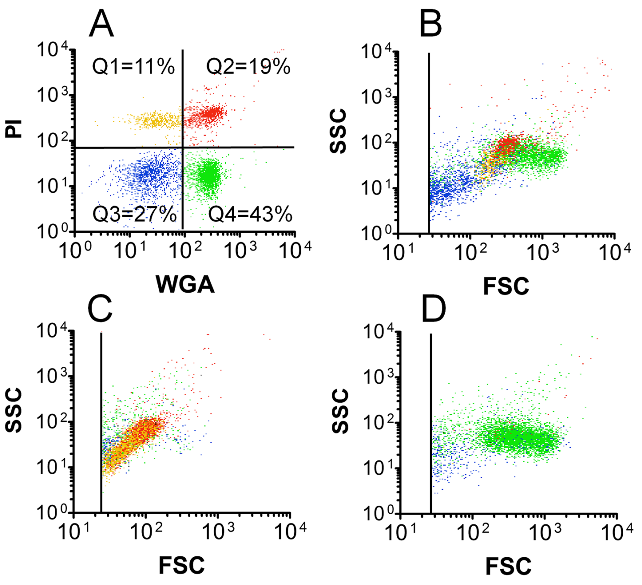

Figure 1. Honey bee hemolymph analysis by flow cytometry. Hemolymph samples from three individual bees were collected and probed with WGA and PI as described in the Methods. Both fluorescence (A) and light scatter (B) data are displayed for hemolymph obtained from a single bee. A) The dot-plot showing fluorescence of WGA-FITC vs PI identifies recognizable groups of particulates in quadrants Q1-Q4, with resulting particle percentages as shown. B) The FSC vs SSC plot provides information on physical parameters for the same particles (in matching color) as those shown in Figure A. Predominance of permeabilized cells (86% of total events from combined Q1 and Q2 quadrants) is shown for a single bee in C, while one bee with 89% plasmatocytes (from Q4) is shown in D.
doi:10.1371/journal.pone.0108486.g001

major particle groups. WGA fluorescence of plasmatocytes was not altered by formalin treatment as described above (data not shown).

Hemolymph from individual bees was often represented by particles in each of the four fluorescence quadrants, such as in the example shown in Figures 1A and B. However, hemolymph occasionally contained an unexpected predominance of events in specific areas of the fluorescence plot. For example, in one bee with a hemocyte profile of 48/38/5/9, 86% of the total events were permeabilized cells (Q1 and Q2) and formed a discrete region (yellow and red events) in the FSC vs SSC plot (Figure 1C). In a different bee with a profile of 0/1/10/89, the majority of the events were plasmatocytes, and produced the predominant population in the FSC vs SSC plot (green events) shown in Figure 1D.

Examination of a total of 634 individual honey bees from different colonies, representing diverse maturation, casteism, condition, and genetic strain, showed a spectrum of particulate profiles. These data provided information to construct a summary plot for FSC vs SSC events (Figure 2A) found in bee hemolymph. This figure shows four regions outlined in colors matching those from the corresponding fluorescence and light scatter plots shown in Figure 1. The summary plot relates properties of particle light scatter (and therefore their physical parameters), which were useful in correlating flow cytometry data to objects seen by microscopy, as described below.

Microscopic analysis of honey bee hemocytes

Hemolymph samples showing a predominance of specific particle types by flow cytometry were examined by microscopy

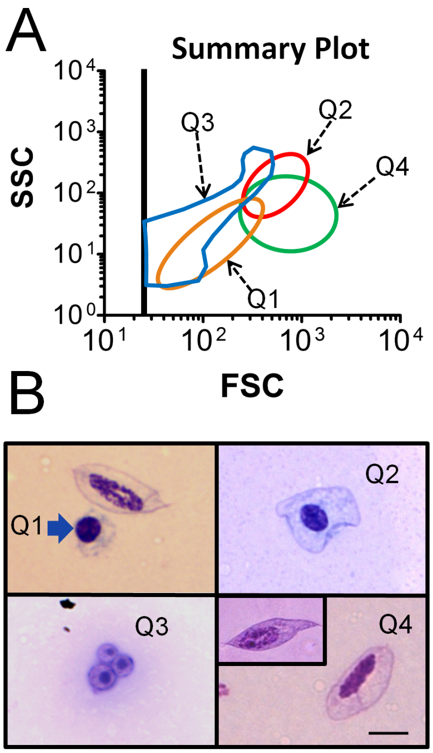

Figure 2. Association of flow cytometry light scatter profiles with particle types observed in Wright-stained smears. A) Analysis of flow cytometry data from a total of 634 individual bees, including several common bee strains, resulted in a FSC vs SSC summary plot that provides information about size and complexity in particles observed in hemolymph. Colors of regions used in the summary plot match those shown in Figure 1. B) Photomicrographs of Wright-stained particles correspond to regions Q1-Q4 of the summary plot include Q1: image of a permeabilized cell (blue arrow) lacking an obvious plasma membrane (and plasmatocyte with plasma membrane for comparison), Q2: permeabilized cell surrounded by a plasma membrane, Q3: microparticles, and Q4: plasmatocyte with rounded termini (and pointed termini, inset). The scale bar represents 5 µm for all images.
doi:10.1371/journal.pone.0108486.g002

following Wright staining as shown in Figure 2B. Events appearing in the Q1 area of Figure 2A corresponded with the type of cell represented in Figure 2B, Q1 identified by the blue arrow (the image also includes a larger membrane-bound plasmatocyte for comparison). Plasma membranes of the Q1 permeabilized cells were typically not visible by Wright stain, and cells usually appeared to contain a diminished amount of cytoplasm. Therefore, dimensions for all hemocyte types were collected only for nuclei. These Q1 permeabilized cells contained round or slightly oval nuclei with a long diameter for the nucleus of 3.99 µm (±0.08 µm, $n = 12$). Flow cytometry data with a predominance of Q2 events corresponded to a different type of permeabilized cell, in which nuclei were indistinguishable in shape and size (3.78 µm±0.12 µm, $n = 12$) from those of Q1. However, Q2 permeabilized cells generally showed evidence of plasma membranes (Figure 2B, Q2). This observation was consistent with Q2 permeabilized cells having greater FSC and SSC values when compared with those found in Q1. Q2 cells showed labeling by the WGA plasma membrane probe. Our data suggested Q1 and Q2 events may represent a continuum of permeabilized cell forms, differing in the amount of associated plasma membrane and cytoplasm, and this was supported by the shift of some cells from

Q2 to Q1 due to formalin treatment, as indicated above. A different type of hemolymph particle (Figure 2B, Q3) appeared too small by microscopy (total particle diameter 2.72 μm ±0.15 μm, n = 12) to represent a complete cell. These acellular (Q3) objects sometimes showed a small darkly-staining central region, as shown in the image. Both the overall cell morphology and nuclei of plasmatocytes (shown in Figure 2B, Q4) were relatively large and elongated with a long dimension for the nucleus of 7.67 μm (±0.22 μm, n = 12). Plasmatocytes invariably appeared to have intact cytoplasm and plasma membranes, and sometimes demonstrated pointed termini (Figure 2B, Q4, inset). The possibility that plasmatocytes exist in two subsets (with rounded or pointed termini) has not been previously reported, but was suggested due to their co-localization in both the fluorescence and light scatter plots. The appearance of intact plasma membranes around plasmatocytes following Wright stain was consistent with their invariant absence of PI staining by flow cytometry.

To further investigate Q1 and Q2 cell permeability and staining by PI, we examined cell samples following incubation with increasing concentrations of saponin. We previously reported that treatment of human neutrophils in 0.01% saponin renders plasma membranes permeable to PI staining [55]. However, PI labeling was not increased in Q1 and Q2 permeabilized cells even after treatment in up to 5% saponin (data not shown). This result supports the notion that permeabilized cell nuclei (but not nuclei of plasmatocytes) were fully accessible to PI without chemical permeabilization of membranes by saponin. This result argues against an intermediate level of PI staining in honey bee hemocytes, as observed in hemocytes from certain lepidopteran species [46].

Examination of hemocytes for evidence of apoptosis

Our results showed honey bee permeabilized cells (but not plasmatocytes) were stained by PI immediately upon collection from the live bee and examination by flow cytometry. This unexpected result suggested the permeabilized cells may be involved in programmed cell death or necrosis. We therefore tested them for evidence of apoptosis using annexin V, which labels exposed phosphatidylserine in cell membranes. We also examined them for evidence of mitochondrial membrane potential using the fluorochrome, JC-1 [56]. Following exposure to PI and annexin V, hemocyte subsets were identified in FSC vs SSC plots as shown in Figure 3A, where permeabilized cells and plasmatocytes were identified using red and blue dots, respectively. Green dots represent cells or microparticles lying outside the specified regions. When fluorescence values for these same events were displayed, permeabilized cells appeared primarily in the upper right quadrant of the plot, indicating labeling by annexin V as well as PI (Figure 3B). These data confirmed accessibility of annexin V to phosphatidylserine in plasma membranes of permeabilized cells, and permeability of plasma membranes to PI. In contrast, plasmatocytes were located mainly in the lower left quadrant, indicating they were not labeled by either PI or annexin V.

Loss of mitochondrial membrane potential revealed by JC-1 staining is typically observed in apoptosis [56]. Hemocytes were therefore probed with JC-1 to identify mitochondrial membrane potential in the presence and absence of the CCCP mitochondrial membrane potential disruptor (Figure 4). Permeabilized cells and plasmatocytes were selected from FSC vs SSC plots as described above, and examined separately to calculate the ratio of the red to green mean fluorescence intensity for the selected cell subsets, which was required to obtain information about mitochondrial membrane potential by JC-1. Unexpectedly, both permeabilized cells and plasmatocytes revealed little if any mitochondrial activity due to their fluorescence values (filled bars), and in both cell types this value paradoxically increased in the presence of the CCCP mitochondrial membrane potential disruptor (open bars). Log-phase murine SP2/0 cells repeatedly showed significantly stronger mitochondrial membrane potential relative to either hemocyte type (P<0.001). Unlike the situation in the hemocytes, the signal in the SP2/0 cells could be significantly (P<0.001) reduced with CCCP.

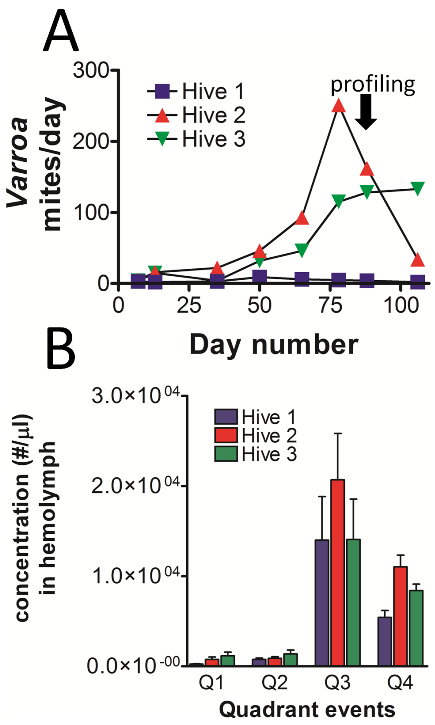

Figure 3. Honey bee hemocyte staining by annexin V. Hemolymph from five bees was pooled and probed with both PI and annexin V, then examined by flow cytometry. A) Events displayed in the FSC vs SSC plot were used to locate regions corresponding to permeabilized cells (red dots) and plasmatocytes (blue dots), where overlap of cell types was minimal. Green dots represent microparticles and cells lying outside the selected regions. B) Fluorescence measurements obtained on particle subsets shown in A (using the same coloring scheme) indicate permeabilized cells are bound by annexin V and PI, while plasmatocytes were not labeled by either fluorochrome. The data are representative of 12 separate examinations involving hemolymph from 60 bees tested on three separate days.
doi:10.1371/journal.pone.0108486.g003

Hemolymph particle identification

Our flow cytometry and microscopy suggested at least three hemocyte subsets and one acellular particle routinely appeared in hemolymph of honey bees. We reasoned that identification of the specific cell types would assist with interpreting their cellular function and role in host defense. However, we found comparison of hemocyte types among different insect species yielded few similarities, as previously reported [26,51]. Nevertheless, we assigned labels to the individual cell subsets observed in the honey bees, based on existing literature. We expect that further refinement of identification for hemolymph particles in honey bees may occur as new molecular, ultrastructural, and functional studies are applied.

We classified both Q1 and Q2 cells (shown in Figure 2B) as permeabilized cells to reflect their unexpected plasma membrane properties. These cells may be derived from granulocytes due to

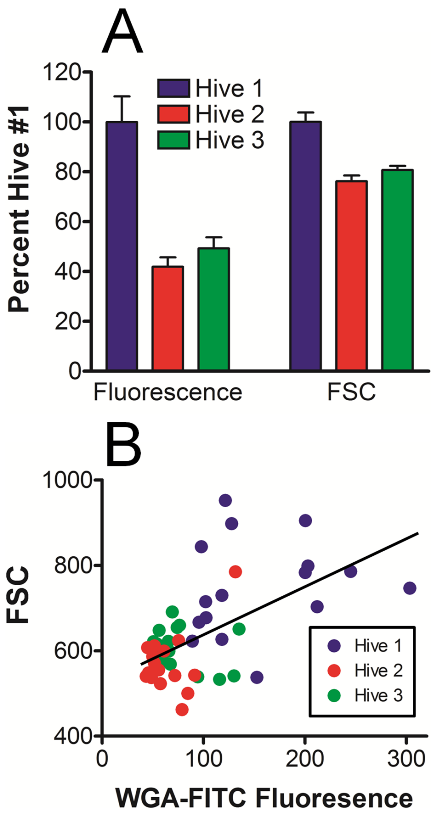

Figure 4. JC-1 staining to identify mitochondrial membrane activity in honey bee hemocytes. Hemolymph samples from five bees were pooled and split into two equal volumes, then exposed to either the JC-1 probe alone, or JC-1 in the presence of the mitochondrial membrane potential disruptor, CCCP. Permeabilized cells and plasmatocytes were identified in FSC vs SSC plots, which were selected to determine the fluorescence intensity for the respective cell type. Bars represent the mean fluorescence intensity ratio (red/green) for replicates, both with (open bars) and without (filled bars) CCCP. Error bars define SEM, n = 7 samples; where each sample represents a pool of five unique bees. The murine SP2/0 cell line was used as a positive control. Data are representative of results obtained on two separate days.
doi:10.1371/journal.pone.0108486.g004

their minimal spreading on glass surface [51] (data not shown), size comparison [26], and labeling with PI [46]. However, the appearance of granularity in these cells after Wright staining was not informative, and their unusual properties warrant further characterization.

When examined by microscopy, Q3 particles in Figure 2B did not show similarity to hemocytes or other cells described, and were typically present in the greatest concentration relative to other hemolymph particles. We typically observed uniformity in shape, but variability in size among these Q3 particles (shown by FSC values, data not shown). We assume these particles are produced as membrane blebs from cells, though the type of cells from which they originate is not yet known. We identified Q3 events as microparticles based on modest similarity to structures described in a variety of mammalian body fluids [57,58]. These objects may be related to microparticles linked with hemolymph clotting in *Drosophila* [59]. At this time, few clues are available to speculate further on the function of these particles in honey bee hemolymph.

Cells shown in Figure 2B, Q4 were identified as plasmatocytes based on labeling by WGA, lack of staining by PI [46], and spindle-shaped morphology in some cells [51]. Though plasmatocytes from *Drosophila* and certain species of Lepidoptera have been observed to spread rapidly on glass [51], we did not observe this response in any cell type in bee hemolymph within 30 minutes at 25°C. Though phagocytosis of foreign objects by plasmatocytes has also been reported by some investigators as reviewed [26], we did not observe this activity in any cell type we observed, despite the observation that free bacteria were occasionally found in hemolymph.

Smears of bee hemolymph sometimes showed hemocyte aggregation, as shown in Figure 5. Such events were typically observed when samples inadvertently contained setae (identified by the white arrow in the inset), which are small feather-like projections from the external surface of the bee. Cell aggregates involved abundant numbers of permeabilized cells as well as some plasmatocytes, which is reminiscent of the encapsulation process by hemocytes in insects, including the lepidopteran species, *Pseudoplusia includens* [60,61].

Possible practical application of hemocyte profiling

We applied hemocyte profiling to bees from three established colonies differing in *Varroa* mite burden, where the mite number collected per day for each hive is represented in Figure 6A. This demonstrated the possibility of hemocyte profiling to explore responses of bees to a specific variable. However, additional colonies would need to be examined to establish specific relationships connecting changes in hemocyte profiles to *Varroa* mite numbers. Except for mite load, the three colonies sampled were similar with regard to strength, location, time since establishment, equipment type, and bee strain. The three colonies differed with regard to numbers of *Varroa* mites, though none had a history of treatment to reduce mite loads. Hemocyte profiling was applied to the three hives on day 88, which was the next sampling opportunity following the peak of mites observed in hive #2. Bees were collected from each colony and hemocyte profile indices were determined as described. The average number of hemolymph particles expressed in units of concentration (#/µl) for Q1-Q4 events is shown in Figure 6B. Significant differences were noted when comparing hives for Q1 (permeabilized cells, P = 0.02

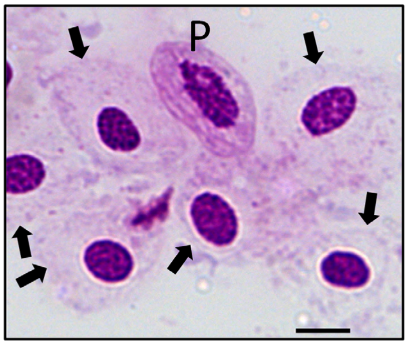

Figure 5. Aggregation of honey bee hemocytes involving permeabilized cells and plasmatocytes. Hemocyte aggregates were occasionally found in Wright-stained smears of hemolymph samples inadvertently containing setae from the surface of the bee (white arrow, inset). Resulting aggregates included both plasmatocytes (plas.) and permeabilized cells (perm.). Plasma membranes were seen surrounding plasmatocytes. This observation contrasted with permeabilized cell nuclei that appeared to be associated with variable amounts of cytoplasm, with little evidence of plasma membranes. The total magnification for each image is 1000×, and the scale bar represents 10 µm for the primary image and 20 µm for the inset.
doi:10.1371/journal.pone.0108486.g005

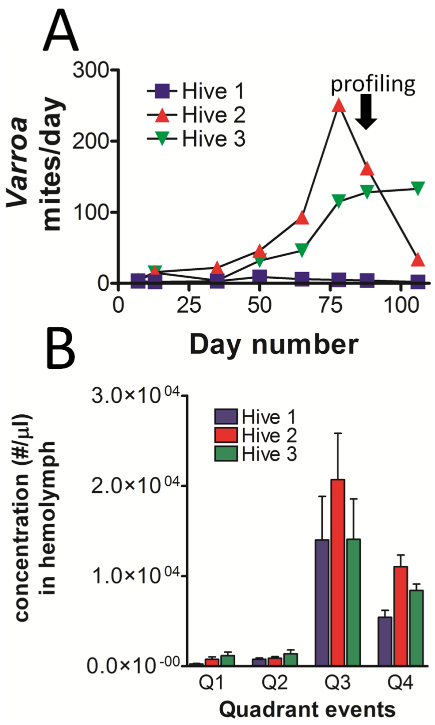

Figure 6. Hemocyte profiling of bees from colonies differing in numbers of *Varroa destructor* mites. A) *Varroa* mites collected from hives 1-3 are represented by the number collected per day on adhesive paper. Hemocyte profiling was performed for all three hives on day 88 following the peak noted in hive 2. B) Hemolymph particles in bees from each hive were quantified and reported in number per µl of hemolymph, according to their localization in specific quadrants (Q1-Q4) of the fluorescence plots. Significant differences between hives were noted for Q1 (permeabilized cells: $P = 0.02$ comparing hive 1 to 3), and for Q4 (plasmatocytes: $P < 0.001$ comparing hive 1 to hive 2). Other comparisons within quadrant types were not found to be significantly different.
doi:10.1371/journal.pone.0108486.g006

comparing hive 1 to 3) and for Q4 (plasmatocytes, $P < 0.001$ when comparing hive 1 to hive 2).

Q1 permeabilized cell and plasmatocyte concentrations were found to be lowest in hive 1, which was parasitized by the fewest number of *Varroa* mites. When comparing the average plasmatocyte number in hemolymph on day 88 to the number of *Varroa* mites recovered per day during the most recent sampling, a correlation coefficient (R^2 value) of 0.995 was observed. These results suggest hemocyte profiles of honey bees may provide information regarding the colony-wide impact of a variable, as well as those within specific members.

Plasmatocyte mean fluorescence intensity due to labeling by WGA and FSC values were found to be significantly greater in hive #1, relative to each of the other two hives on day 88 ($P < 0.001$ for both WGA and FSC, Figure 7A). To further investigate the relationship between WGA labeling and plasmatocyte size, the mean green fluorescence intensity was compared with FSC values by scatter plot for each of the bees from hives 1–3. Each symbol was used to represent the average plasmatocyte value from a single bee, and was color matched to the respective hive (Figure 7B). Plasmatocytes from hives 1–3 collectively showed a positive correlation ($R^2 = 0.33$, $P < 0.001$) between WGA fluorescence and cell size (FSC). These results indicated that plasmatocytes in bees from hive #1 were significantly larger and more fluorescent

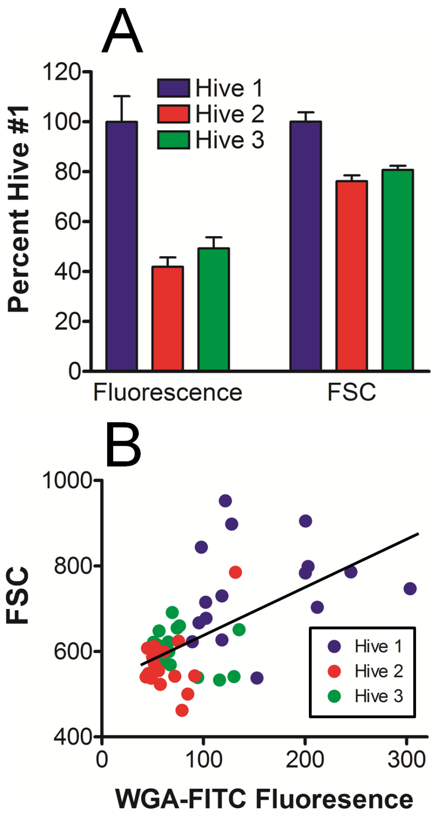

Figure 7. Correlation of plasmatocyte WGA-FITC fluorescence to FSC in bees exposed to variable *Varroa* mite numbers. A) Plasmatocyte populations were selected from FSC vs SSC plots for bees represented in Figure 6B, then evaluated for both WGA-FITC fluorescence and FSC with respect to each hive. Plasmatocytes from bees in hive 1 showed significantly greater ($P < 0.001$, $n = 16$) WGA-FITC labeling intensity and FSC values when compared to either hives 2 or 3. By contrast, plasmatocytes from hives 2 and 3 were not significantly different ($P > 0.05$) for either parameter. B) Linear regression analysis for plasmatocytes from all hives collectively shows correlation ($R^2 = 0.33$, $P < 0.001$) of WGA-FITC labeling to FSC values, where color labeling of individual bees identifies the hive number.
doi:10.1371/journal.pone.0108486.g007

(though lower in concentration) than those obtained from the other two hives.

Discussion

Honey bee hemocyte profile analysis by flow cytometry

A worldwide effort is underway to mitigate global losses of honey bees, though a comprehensive understanding of this problem has proven elusive. Current evidence indicates *Varroa* mites contribute to colony loss, as mites may induce immune suppression in bees [62], as well as transmit viral pathogens [18,63–65]. Bacterial infection may further predispose immune deficiency in honey bees [10,62]. In spite of this situation, relatively few studies are focused on cellular defense mechanisms in honey bees. These concerns suggest a need for better strategies to monitor hemocyte subsets in honey bees and to better define the contribution of pathogens in colony failure.

The hemocyte profiling method we describe can complement functional, cytological, or molecular analyses in honey bees, and provide information with resolution to a single bee. A flow cytometer is needed for the analysis, but hemocyte profiling is

rapid and does not otherwise require specialized equipment, laborious methods, or expensive reagents. Our typical procedure involving WGA and PI staining did not involve centrifugation of samples at any point, thereby avoiding cell loss and possible sedimentation artifacts in cells. Flow cytometry can quickly provide large data sets from small sample sizes. We found that 2–3 µl of hemolymph is sufficient for both smear preparation and flow cytometry for a single bee. Modern flow cytometry software that utilizes data analysis functions to generate heat maps can relate sample groups with specific trends shown by a number of different measurements.

Our results revealed unexpected diversity in hemocyte profiles of honey bees within a single hive. We found examples of individuals with profiles that were dramatically enriched for specific quadrant types when plotted by fluorescence due to PI and WGA. One possible reason for the observed variation could be age-related changes in hemocyte types that respond normally to temporal influences in the adult insect. Such differences could impact changes in bee immunity, which has been a subject of considerable interest. Dynamics of hemocyte profiles may also reflect altered conditions within the colony, external influences such as weather or chemical exposure, or responses to infectious agents.

Plasma membrane differences in permeabilized cells

The notion that permeabilized cell plasma membranes are indeed compromised was supported by several independent observations in our study. The first is that their nuclei are uniformly accessible to PI, which is frequently used in the analysis of mammalian cells to identify dead or apoptotic cells and those with permeabilized plasma membranes. Staining of cells by PI has been noted in other insect species [45,46], but the reason for this observation has not been extensively explored. The second is that the Q1 permeabilized cells were not labeled by the plasma membrane probe, WGA (though Q2 permeabilized cells were bound by that probe). Finally, we noted that plasma membranes are difficult to observe surrounding Q1 permeabilized cells when examined by microscopy following Wright stain. These observations may be related to a previous report of "cytoplasm disruption" in granulocytes from the stingless bee, *Melipona scutellaris* [54], which shares the Hymenoptera order with the honey bee.

We cannot exclude the possibility that the microparticles we described are derived from membrane blebs produced by permeabilized cells undergoing apoptosis. This activity is seen in mycopathogen-induced apoptosis in hemocytes of the pine beetle (*Dendrolimus tabulaeformis*) larvae [66], and in apoptotic hemocytes from wax moth (*Galleria mellonella*) infected with the bacterium, *Pseudomonas aeruginosa* [67].

Hemocyte apoptosis?

We utilized flow cytometry to show that honey bee permeabilized cells were bound by annexin V, consistent with the possibility that they are undergoing apoptosis. However, permeabilized cells also label with PI. Thus, it is possible that plasma membrane permeability renders them accessible to annexin V by exposing phosphatidylserine on the intracellular aspect of the cell membrane, rather than on the membrane exterior which would be consistent with apoptosis. As such, we cannot definitively conclude that annexin V labeling of permeabilized cells is due to apoptosis and not necrosis caused by some other factor. However, the lack of annexin V binding on plasmatocytes argues against apoptosis in that cell type.

Our results using the JC-1 probe indicated an apparent lack of mitochondrial membrane potential both in permeabilized cells and plasmatocytes of honey bees. This same approach was successful showing mitochondrial activity in our cultured mouse myeloma cells, in which the signal was significantly reduced using the CCCP mitochondrial membrane potential disruptor. This information could indicate that examination of mitochondrial activity in honey bee hemocytes is not feasible with JC-1. Yet this staining method was used to show mitochondrial activity in hemocytes of lepidopteran larvae [52]. Another explanation consistent with our data, is that the bees we examined contained hemocytes with inactive mitochondria. Further analyses are needed to investigate this possibility, and to determine whether this condition is associated with immune deficiency in honey bees.

Our data collectively indicate the permeabilized cells we observed in honey bee hemolymph are either dead or damaged. Their condition suggests they may be unlike typical classes of invertebrate hemocytes that have been described [26,51]. In mammals, activation of neutrophils can lead to liberation of microbicidal oxygen radicals that lead to cell death. This activity can result in production of neutrophil extracellular traps (NETS), which have received considerable attention since they were first described in 2004 [68]. Whether the permeabilized hemocytes of bee hemolymph we observed bear evidence of caustic antimicrobial activity, specialized defensive mechanism, or natural progression toward elimination and removal, is unclear at this time.

Hemocyte aggregation

Despite absence of obvious mitochondrial membrane potential in honey bee hemocytes, we found that these cells appear to aggregate upon contact with the external surface of the bee. This suggests a natural mechanism to suppress hemolymph loss following cuticle damage, as indicated by electron microscopy [69]. Resulting cell clotting may also be similar to hemocyte encapsulation in response to inflammatory stimuli [61], pathogen attack, or another physiological trigger. Therefore, bee permeabilized cells and plasmatocytes appear to display a molecular ligature for binding to other cells or surfaces; and through this mechanism, a functional role in response to pathogens or wound healing.

A possible application of hemocyte profiling

Our results indicated an external stress factor such as *Varroa* mite burden may influence hemocyte profiles in honey bees. The changes we observed were seen both in the numbers of permeabilized cells and plasmatocytes, as well as in the size and labeling intensity of plasmatocytes by WGA. Hemocyte profile plasticity may therefore be utilized as a means of evaluating certain aspects of insect physiology in response to external conditions as suggested [32]. To our knowledge, size differences in hemocytes have not previously been suggested to be a measure of physiological changes in hymenopteran insects.

The possibility that certain aspects of honey bee hematopoesis are affected by external stimuli identifies an opportunity to further examine this aspect of bee immunity. Hematological evidence in mammals shows the size of certain cells can be a clue to specific diseases or nutritional deficiencies. For example, human red blood cells appearing smaller or larger than normal (microcytic or macrocytic conditions) can be associated with iron or folate/vitamin B12 deficiency, respectively [70]. Whether the size of plasmatocytes in honey bees can be linked with a specific condition associated with colony health is not currently known.

Conclusion

In summary, we report a rapid method of examining honey bee hemocyte profiles that may be sensitive to conditions that impact their health and social structure. Expansion of this approach to connect indices of honey bee hemolymph with stress factors will provide a better understanding of their susceptibility to challenge, disease, and hive failure. Further studies are needed to unravel these complex relationships.

Acknowledgments

We appreciate the honey bee hemolymph sample collection and assistance with analyses by Johana Teigen and Samantha Fredrickson and undergraduate students in the Applied Science Program at the University of Wisconsin-Stout. The authors acknowledge Kim and Brad Mogen and Karen Klyczek of University of Wisconsin-River Falls for collaboration in bee collection and helpful perspectives on data interpretation, statistical assistance provided by Jim Church, and review of the manuscript by Randy Daughters.

Author Contributions

Conceived and designed the experiments: JB NB WM. Performed the experiments: WM JB MK. Analyzed the data: WM NB JB MK. Contributed reagents/materials/analysis tools: WM JB NB. Wrote the paper: WM NB JB.

References

1. Calderone NW (2012) Insect pollinated crops, insect pollinators and US agriculture: trend analysis of aggregate data for the period 1992–2009. PLoS One 7: e37235.
2. Steinhauer NA, Rennich K, Wilson ME, Caron DM, Lengerich EJ, et al. (2014) A national survey of managed honey bee 2012–2013 annual colony losses in the USA: results from the Bee Informed Partnership. J Apicul Res 53: 1–18.
3. Dainat B, Vanengelsdorp D, Neumann P (2012) Colony collapse disorder in Europe. Environ Microbiol Rep 4: 123–125.
4. Pirk CWW, Human H, Crewe RM, Vanengelsdorp D (2014) A survey of managed honey bee colony losses in the Republic of South Africa-2009 to 2011. J Apicul Res 53: 35–42.
5. Vanengelsdorp D, Speybroeck N, Evans JD, Nguyen BK, Mullin C, et al. (2010) Weighing risk factors associated with bee colony collapse disorder by classification and regression tree analysis. J Econ Entomol 103: 1517–1523.
6. Rosenkranz P, Aumeier P, Ziegelmann B (2010) Biology and control of Varroa destructor. J Invertebr Pathol 103 Suppl 1: S96–119.
7. McMullan JB, Brown MJ (2009) A qualitative model of mortality in honey bee (Apis mellifera) colonies infested with tracheal mites (Acarapis woodi). Exp Appl Acarol 47: 225–234.
8. Higes M, Meana A, Bartolome C, Botias C, Martin-Hernandez R (2013) Nosema ceranae (Microsporidia), a controversial 21st century honey bee pathogen. Environ Microbiol Rep 5: 17–29.
9. Chen Y, Pettis JS, Zhao Y, Liu X, Tallon LJ, et al. (2013) Genome sequencing and comparative genomics of honey bee microsporidia, Nosema apis reveal novel insights into host-parasite interactions. BMC Genomics 14: 451.
10. Gatschenberger H, Azzami K, Tautz J, Beier H (2013) Antibacterial immune competence of honey bees (Apis mellifera) is adapted to different life stages and environmental risks. PLoS One 8: e66415.
11. Foley K, Fazio G, Jensen AB, Hughes WO (2012) Nutritional limitation and resistance to opportunistic Aspergillus parasites in honey bee larvae. J Invertebr Pathol 111: 68–73.
12. Runckel C, Flenniken ML, Engel JC, Ruby JG, Ganem D, et al. (2011) Temporal analysis of the honey bee microbiome reveals four novel viruses and seasonal prevalence of known viruses, Nosema, and Crithidia. PLoS One 6: e20656.
13. Chen Y, Zhao Y, Hammond J, Hsu HT, Evans J, et al. (2004) Multiple virus infections in the honey bee and genome divergence of honey bee viruses. J Invertebr Pathol 87: 84–93.
14. Genersch E, Aubert M (2010) Emerging and re-emerging viruses of the honey bee (Apis mellifera L.). Vet Res 41: 54.
15. Vanengelsdorp D, Evans JD, Saegerman C, Mullin C, Haubruge E, et al. (2009) Colony collapse disorder: a descriptive study. PLoS One 4: e6481.
16. Evans JD, Schwarz RS (2011) Bees brought to their knees: microbes affecting honey bee health. Trends Microbiol 19: 614–620.
17. Cornman RS, Tarpy DR, Chen Y, Jeffreys L, Lopez D, et al. (2012) Pathogen webs in collapsing honey bee colonies. PLoS One 7: e43562.
18. Dainat B, Evans JD, Chen YP, Gauthier L, Neumann P (2012) Predictive markers of honey bee colony collapse. PLoS One 7: e32151.
19. Pettis JS, Lichtenberg EM, Andree M, Stitzinger J, Rose R, et al. (2013) Crop Pollination Exposes Honey Bees to Pesticides Which Alters Their Susceptibility to the Gut Pathogen Nosema ceranae. PLoS One 8: e70182.
20. Mullin CA, Frazier M, Frazier JL, Ashcraft S, Simonds R, et al. (2010) High levels of miticides and agrochemicals in North American apiaries: implications for honey bee health. PLoS One 5: e9754.
21. Di PG, Cavaliere V, Annoscia D, Varricchio P, Caprio E, et al. (2013) Neonicotinoid clothianidin adversely affects insect immunity and promotes replication of a viral pathogen in honey bees. Proc Natl Acad Sci U S A 110: 18466–18471.
22. Di PG, Salignon M, Le CY, Belzunces LP, Decourtye A, et al. (2013) Influence of pollen nutrition on honey bee health: do pollen quality and diversity matter? PLoS One 8: e72016.
23. Vanengelsdorp D, Tarpy DR, Lengerich EJ, Pettis JS (2013) Idiopathic brood disease syndrome and queen events as precursors of colony mortality in migratory beekeeping operations in the eastern United States. Prev Vet Med 108: 225–233.
24. Vilmos P, Kurucz E (1998) Insect immunity: evolutionary roots of the mammalian innate immune system. Immunol Lett 62: 59–66.
25. Evans JD, Aronstein K, Chen YP, Hetru C, Imler JL, et al. (2006) Immune pathways and defence mechanisms in honey bees Apis mellifera. Insect Mol Biol 15: 645–656.
26. Lavine MD, Strand MR (2002) Insect hemocytes and their role in immunity. Insect Biochem Mol Biol 32: 1295–1309.
27. Marmaras VJ, Lampropoulou M (2009) Regulators and signalling in insect haemocyte immunity. Cell Signal 21: 186–195.
28. de Graaf DC, Dauwe R, Walravens K, Jacobs FJ (2002) Flow cytometric analysis of lectin-stained haemocytes of the honeybee (Apis mellifera). Apidologie 33: 571–579.
29. Schmid MR, Brockmann A, Pirk CW, Stanley DW, Tautz J (2008) Adult honeybees (Apis mellifera L.) abandon hemocytic, but not phenoloxidase-based immunity. J Insect Physiol 54: 439–444.
30. Amdam GV, Aase AL, Seehuus SC, Kim FM, Norberg K, et al. (2005) Social reversal of immunosenescence in honey bee workers. Exp Gerontol 40: 939–947.
31. Wilson-Rich N, Dres ST, Starks PT (2008) The ontogeny of immunity: development of innate immune strength in the honey bee (Apis mellifera). J Insect Physiol 54: 1392–1399.
32. Amdam GV, Hartfelder K, Norberg K, Hagen A, Omholt SW (2004) Altered physiology in worker honey bees (Hymenoptera: Apidae) infested with the mite Varroa destructor (Acari: Varroidae): a factor in colony loss during overwintering? J Econ Entomol 97: 741–747.
33. Niu L, Ma Y, Mannakkara A, Zhao Y, Ma W, et al. (2013) Impact of single and stacked insect-resistant Bt-cotton on the honey bee and silkworm. PLoS One 8: e72988.
34. Gilliam M, Shimanuki H (1967) In vitro phagocytosis of Nosema apis spores by honey-bee hemocytes. J Invertebr Pathol 9: 387–389.
35. Flenniken ML, Andino R (2013) Non-specific dsRNA-mediated antiviral response in the honey bee. PLoS One 8: e77263.
36. Evans JD, Spivak M (2010) Socialized medicine: individual and communal disease barriers in honey bees. J Invertebr Pathol 103 Suppl 1: S62–S72.
37. Chan QW, Melathopoulos AP, Pernal SF, Foster LJ (2009) The innate immune and systemic response in honey bees to a bacterial pathogen, Paenibacillus larvae. BMC Genomics 10: 387.
38. Harpur BA, Zayed A (2013) Accelerated evolution of innate immunity proteins in social insects: adaptive evolution or relaxed constraint? Mol Biol Evol 30: 1665–1674.
39. Bull JC, Ryabov EV, Prince G, Mead A, Zhang C, et al. (2012) A strong immune response in young adult honeybees masks their increased susceptibility to infection compared to older bees. PLoS Pathog 8: e1003083.
40. Munch D, Amdam GV, Wolschin F (2008) Ageing in a eusocial insect: molecular and physiological characteristics of life span plasticity in the honey bee. Funct Ecol 22: 407–421.
41. Jefferson JM, Dolstad HA, Sivalingam MD, Snow JW (2013) Barrier immune effectors are maintained during transition from nurse to forager in the honey bee. PLoS One 8: e54097.
42. Williams MJ (2007) Drosophila hemopoiesis and cellular immunity. J Immunol 178: 4711–4716.
43. Oliver JD, Dusty LJ, Parikh G, Bartholomay L (2011) Comparative analysis of hemocyte phagocytosis between six species of arthropods as measured by flow cytometry. J Invertebr Pathol 108: 126–130.
44. Ling E, Shirai K, Kanehatsu R, Kiguchi K (2005) Reexamination of phenoloxidase in larval circulating hemocytes of the silkworm, Bombyx mori. Tissue Cell 37: 101–107.
45. Ling E, Shirai K, Kanekatsu R, Kiguchi K (2003) Classification of larval circulating hemocytes of the silkworm, Bombyx mori, by acridine orange and propidium iodide staining. Histochem Cell Biol 120: 505–511.

46. Nakahara Y, Shimura S, Ueno C, Kanamori Y, Mita K, et al. (2009) Purification and characterization of silkworm hemocytes by flow cytometry. Dev Comp Immunol 33: 439–448.
47. Yue F, Zhou Z, Wang L, Wang M, Song L (2014) A conserved zinc finger transcription factor GATA involving in the hemocyte production of scallop Chlamys farreri. Fish Shellfish Immunol.
48. Kuhn-Nentwig L, Kopp LS, Nentwig W, Haenni B, Streitberger K, et al. (2014) Functional differentiation of spider hemocytes by light and transmission electron microscopy, and MALDI-MS-imaging. Dev Comp Immunol 43: 59–67.
49. Shi XZ, Wang L, Xu S, Zhang XW, Zhao XF, et al. (2014) A galectin from the kuruma shrimp (Marsupenaeus japonicus) functions as an opsonin and promotes bacterial clearance from hemolymph. PLoS One 9: e91794.
50. de Barros CM, Emrich LC, Mello AA, da Fonseca RN, Allodi S (2014) Regulation of nitric-oxide production in hemocytes of the ascidian Phallusia nigra. Nitric Oxide 38: 26–36.
51. Ribeiro C, Brehelin M (2006) Insect haemocytes: what type of cell is that? J Insect Physiol 52: 417–429.
52. Suderman RJ, Pruijssers AJ, Strand MR (2008) Protein tyrosine phosphatase-H2 from a polydnavirus induces apoptosis of insect cells. J Gen Virol 89: 1411–1420.
53. Human H, Brodschneider R, Dietemann V, Dively G, Ellis J, et al. (2013) Miscellaneous Standard Methods for Apis mellifera Research. J Apicul Res 52.
54. Amaral IM, Moreira Neto JF, Pereira GB, Franco MB, Beletti ME, et al. (2010) Circulating hemocytes from larvae of Melipona scutellaris (Hymenoptera, Apidae, Meliponini): cell types and their role in phagocytosis. Micron 41: 123–129.
55. Burritt JB, Quinn MT, Jutila MA, Bond CW, Jesaitis AJ (1995) Topological mapping of neutrophil cytochrome b epitopes with phage-display libraries. J Biol Chem 270: 16974–16980.
56. Cossarizza A, Salvioli S (2001) Flow cytometric analysis of mitochondrial membrane potential using JC-1. Curr Protoc Cytom Chapter 9: Unit.
57. Roseblade A, Luk F, Rawling T, Ung A, Grau GE, et al. (2013) Cell-derived microparticles: new targets in the therapeutic management of disease. J Pharm Pharm Sci 16: 238–253.
58. Jy W, Horstman LL, Ahn YS (2010) Microparticle size and its relation to composition, functional activity, and clinical significance. Semin Thromb Hemost 36: 876–880.
59. Schmidt O, Theopold U (1997) Helix pomatia lectin and annexin V, two molecular probes for insect microparticles: possible involvement in hemolymph coagulation. J Insect Physiol 43: 667–674.
60. Pech LL, Strand MR (1996) Granular cells are required for encapsulation of foreign targets by insect haemocytes. J Cell Sci 109(Pt 8): 2053–2060.
61. Lavine MD, Strand MR (2001) Surface characteristics of foreign targets that elicit an encapsulation response by the moth Pseudoplusia includens. J Insect Physiol 47: 965–974.
62. Yang X, Cox-Foster DL (2005) Impact of an ectoparasite on the immunity and pathology of an invertebrate: evidence for host immunosuppression and viral amplification. Proc Natl Acad Sci U S A 102: 7470–7475.
63. Yang X, Cox-Foster D (2007) Effects of parasitization by Varroa destructor on survivorship and physiological traits of Apis mellifera in correlation with viral incidence and microbial challenge. Parasitology 134: 405–412.
64. Shen M, Yang X, Cox-Foster D, Cui L (2005) The role of varroa mites in infections of Kashmir bee virus (KBV) and deformed wing virus (DWV) in honey bees. Virology 342: 141–149.
65. Francis RM, Nielsen SL, Kryger P (2013) Varroa-virus interaction in collapsing honey bee colonies. PLoS One 8: e57540.
66. Fan J, Xie Y, Xue J, Zhang Y, Yang Q (2013) Cellular apoptosis of hemocytes from Dendrolimus tabulaeformis Tsai et Liu larvae induced with the secondary metabolites of Beauveria brongniartii (Sacc.) Petch. PLoS One 8: e71600.
67. Mizerska-Dudka M, Andrejko M (2014) Galleria mellonella hemocytes destruction after infection with Pseudomonas aeruginosa. J Basic Microbiol 54: 232–246.
68. Brinkmann V, Reichard U, Goosmann C, Fauler B, Uhlemann Y, et al. (2004) Neutrophil extracellular traps kill bacteria. Science 303: 1532–1535.
69. Kanbar G, Engels W (2003) Ultrastructure and bacterial infection of wounds in honey bee (Apis mellifera) pupae punctured by Varroa mites. Parasitol Res 90: 349–354.
70. Ford J (2013) Red blood cell morphology. Int J Lab Hematol 35: 351–357.
